# Alternative Protein Sources and Novel Foods: Benefits, Food Applications and Safety Issues

**DOI:** 10.3390/nu15061509

**Published:** 2023-03-21

**Authors:** Laura Quintieri, Chiara Nitride, Elisabetta De Angelis, Antonella Lamonaca, Rosa Pilolli, Francesco Russo, Linda Monaci

**Affiliations:** 1 Institute of Sciences of Food Production, National Research Council of Italy (ISPA-CNR), Via Giovanni Amendola 122/O, 70126 Bari, Italy; 2 Department of Agricultural Sciences, University of Naples Federico II, Via Università 100, 80055 Portici, Italy; 3 Functional Gastrointestinal Disorders Research Group, National Institute of Gastroenterology IRCCS “Saverio de Bellis”, 70013 Castellana Grotte, Italy

**Keywords:** underutilized legumes, fungi, insects, aquatic weeds, bioactive peptides, food formulation, novel foods, alternative proteins

## Abstract

The increasing size of the human population and the shortage of highly valuable proteinaceous ingredients has prompted the international community to scout for new, sustainable, and natural protein resources from invertebrates (e.g., insects) and underutilized legume crops, unexploited terrestrial and aquatic weeds, and fungi. Insect proteins are known for their nutritional value, being rich in proteins with a good balance of essential amino acids and being a valuable source of essential fatty acids and trace elements. Unconventional legume crops were found rich in nutritional, phytochemical, and therapeutic properties, showing excellent abilities to survive extreme environmental conditions. This review evaluates the recent state of underutilized legume crops, aquatic weeds, fungi, and insects intended as alternative protein sources, from ingredient production to their incorporation in food products, including their food formulations and the functional characteristics of alternative plant-based proteins and edible insect proteins as novel foods. Emphasis is also placed on safety issues due to the presence of anti-nutritional factors and allergenic proteins in insects and/or underutilized legumes. The functional and biological activities of protein hydrolysates from different protein sources are reviewed, along with bioactive peptides displaying antihypertensive, antioxidant, antidiabetic, and/or antimicrobial activity. Due to the healthy properties of these foods for the high abundance of bioactive peptides and phytochemicals, more consumers are expected to turn to vegetarianism or veganism in the future, and the increasing demand for such products will be a challenge for the future.

## 1. Introduction

In the last years, the increase in the human population and depletion of natural resources have become among the most critical issues to be faced worldwide. The current world population comprises 7.5 billion people and is foreseen to increase up to 9–10 billion by 2050 [1]. As a result, the demand for sustainable, healthy, and nourishing foods is constantly raising. Nowadays, food systems represent the major contributors to environmental issues such as global greenhouse gas (GHG) emissions, deforestation, and water consumption [2,3]. Meat production has been found to have a dramatic impact on GHG emissions and consumption of water [4]. Thus, alternative sources of valuable proteins are urgently needed.

For a long time, plant proteins were deemed to possess a minor nutritional value compared to meat proteins, even though this trend is recently reversing [5]. As plant proteins come along with other plant ingredients and accompanying nutrients, a direct comparison with meat proteins in terms of overall benefit has been hardly achieved. A product with over 30% protein content (dry matter) and low-fat content is considered an excellent meat substitute [6]. It is also worth noting that substitutes or alternatives to meat products should be characterized by their similarity to meat protein digestibility-corrected amino acid score (PDCAAS) [7,8].

The technological functions of proteins are linked to their origin and concentration in the food ingredient. Based on their functioning properties, such as water-binding capacity, many proteins can be used in preparing of meat alternatives. Typical proteins exploited for this purpose are pea protein isolate, soy protein isolate, wheat gluten blends, etc. [9]. Gluten proteins, and containing glutenins and gliadins, are used to prepare meat alternatives, imparting the chewiness and structure to the products [10]. Seitan is a pure gluten of meat alternative that is largely consumed in vegetarian and vegan diets and can be found in the form of burgers, sausages, and nuggets seasoned and colored, for example, using microbial natural pigments. Similar to seitan, tofu is a product of thermal coagulation of soybean proteins promoted by calcium ions, which is a versatile alternative to dairy products. On the other side, drawbacks in using gluten for meat alternative analogues rely on the allergenic potential towards allergic individuals and people suffering from celiac disease, limiting its use. In addition to allergic effects, the addition of alternative proteins such as soy proteins to food formulations confer bitter and astringent flavor, limiting the consumer’s acceptability. Thus, novel sources of proteins are to be investigated. Fungi-based meat alternatives look and taste just like meat, and their environmental impact is next to nothing [11]; proteins from edible fungi are also attracting interest for their healthy nutritional profile [12,13]. On the other side, insect proteins are also exploited in food production since they show good oil and water retention capacity, emulsion, and foaming activities [14]. Several proteins derived from legumes represent a good choice for their gel-forming abilities, crucial for immobilizing fat and entrapping water within the matrix of emulsion-type alternative protein products [15].

Plant-based products, such as vegetables and pulses, have gained increasing attention over the time, representing an excellent source of proteins and phytochemicals. In this regard, it has been documented that diets rich in these products may prevent the induction of a wide range of diseases [16]. Over the last decade, the re-introduction of underutilized (neglected, minor, or orphan) crops were found to be an excellent strategy to improve global food security [17]. Underutilized crops are referred to species cultivated in their centers of origins but less used at the global level because they are non-competitive in terms of agronomic, economic, or cultural properties compared to those most cultivated. They are rich in nutritional, phytochemical, and therapeutic qualities [17]. In addition, their ability to survive extreme environmental conditions makes these species a good choice to face climate change and the vulnerability of agriculture and horticulture [18]. Alongside these categories of protein sources, leaves also represent a valuable source of plant proteins to be implemented in the formulation of meat alternatives. Dried powder obtained from fresh leaves can contain up to 30% of the protein content. Beyond terrestrial panacea plants, some aquatic weed species such as duckweed are superfood highly consumed in Asia with a high protein content attracting enormous interest as alternative protein sources to be used in human nutrition.

Insects are a common food for 2 billion people across the globe [19], and over 2000 edible species are known. Because of their high protein content and nutritional value, edible insects, especially crickets, are currently considered a solution to the growing protein demand worldwide [20,21]. Despite its widespread consumption in eastern countries, the European market appears to convert to such new eating habit based on insect foods. However, the growing need for alternative sources of proteins other than meat and their sustainable growing conditions is promoting their acceptance in western countries for the several advantages offer. Considering the urgent need for alternative sources of proteins, on 3 January 2023, the European Union allowed the placing on the market of *Acheta domesticus*, i.e., the domestic cricket, in partially defatted powder as a novel food [22]; yellow mealworm and migratory locus were previously approved [23]. Most insects are high in micronutrients such as potassium, calcium, iron, magnesium, and selenium and are also a good source of proteins [24]. An overview of the evolution of the European legislation issued on novel food is reported in Table 1.

In most cases, they have higher quality proteins than plant and meat proteins, in terms of their nutritional value, total protein content, essential amino acid composition, and protein efficiency [25]. According to studies conducted by Mason et al. [19], the production of one gram of beef requires 21 times more water than the production of the same amount of protein from cricket, making this protein source a valuable and sustainable alternative to the conventional meat-based proteins [26,27]. Insects have great biodiversity and biomass, potentially being a natural resource to produce new bioactive peptides [28]. Several studies show that insect peptides/polypeptides have antihypertensive, antioxidant, antidiabetic, and antimicrobial activities [21,29,30,31]. Biotechnology appears to be a promising approach to mass production of insect bioactive peptides. A risk of triggering allergic reactions based on cross-allergenic reactions to crustacean and house dust mite proteins is likely to occur in sensitive individuals [32,33].

**Table 1:** Chronologically ordered set of the released legal documents regarding insect production for food and feed.

Name of the Document	Year	Reference
Regulation (EU) 2015/2283 of the European Parliament and of the Council of 25 November 2015 on novel foods, amending Regulation (EU) No. 1169/2011 of the European Parliament and of the Council and repealing Regulation (EC) No. 258/97 of the European Parliament and of the Council and Commission Regulation (EC) No. 1852/2001.	2015	[34]
EFSA Scientific Opinion “Risk profile related to the production and consumption of insects as food and feed” issued 8 October 2015.	2015	[35]
IPIFF information document “Regulation (EU) 2015/2283 on novel foods- Briefing paper on the provisions relevant to the commercialisation of insect-based products intended for human consumption in the EU."	2015	[36]
Commission Regulation (EU) 2017/893 of 24 May 2017 amending Annexes I and IV to Regulation (EC) No. 999/2001 of the European Parliament and of the Council and Annexes X, XIV and XV to Commission Regulation (EU) No. 142/2011 as regards the provisions on processed animal protein.	2017	[37]
Commission Implementing Regulation (EU) 2017/2469 of 20 December 2017 laying down administrative and scientific requirements for applications referred to in Article 10 of Regulation (EU) 2015/2283 of the European Parliament and of the Council on novel foods.	2017	[38]
‘Novel Food’ Report: Opinion on the Risk Profile for House Cricket (*Acheta domesticus*) by the Swedish University of Agricultural Sciences (EFSA funded report, adopted on 6 July 2018).	2018	[39]

This review aims to explore novel alternative proteins to meat, including underutilized legumes, aquatic weeds, edible fungi, and insects. The food applications and functional activities related to their derived peptides will also be highlighted in order to underline recent advances and shed light on unexplored fields. In the last section, safety issues related to the introduction of some novel foods in the market will also be discussed.

## 2. Underutilized Legume Crops

**Figure 1:**
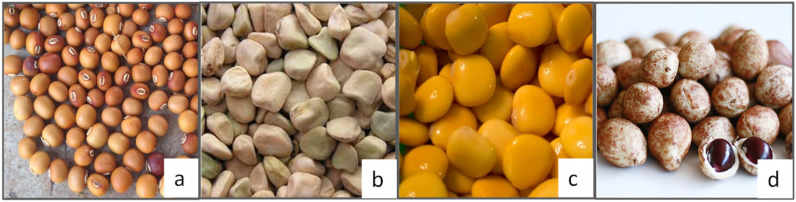
Underutilized legumes: (**a**) Winged beans (**b**) Grasspea (**c**) Lupins (**d**) Bambara groundnut.

In plant realm, *Fabaceae* (*Leguminosae*) family is a source of seeds known as pulses, showing the best compromise between sustainability and nutritional value, in terms of dietary proteins and other essential nutrients, especially in countries where animal protein sources are scarce and considered expensive or avoided for religious reasons (i.e., India) [40,41]. Despite the high number of legume species currently known, only some show well-established domestication and cultivation, such as soybean (*Glycine max* L.), cowpea (*Vigna unguiculata* L.), peanut (*Arachis hypogaea* L.), common beans (*Phaseolus vulgaris* L.), pea (*Pisum sativum* L.), lentil (*Lens culinaris*), and chicken pea (*Cicer arietinum* L.). Other legumes less known and less exploited are counted among the underutilized and orphan species. This category includes grass pea (*Lathyrus sativus* L.), lupine (*Lupinus Albus)*, winged bean *(Psophocarpus tetragonolobus*), and bambara groundnut (*Vigna subterranea*), shown in Figure 1, which are considered underutilized legumes with promising potentials because of their hardiness, remarkable nutritional profiles, and desirable protein content, especially in their seeds. Orphan legumes that are comparable with soybean in terms of nutritional value are described.

Grass pea (*Lathyrus sativus* L.) is a member of the *Fabaceae* (*Leguminosae*) family, subfamily *Papilionoideae*, tribe *Vicieae,* representing an important annual legume crop cultivated in several areas of the world in South, Southeast Asia, Middle East, Eastern Europe, North America, South America, and East Africa [42,43], with production estimated at 1.2 million tons [44]. Grass pea seeds are used as a pulse, *dahl,* and flour to prepare different savories, sweets, and snacks. Seeds contain about 8.6–34.6% proteins, higher than chickpea (18%), field pea (21%), French bean (20%), and similar to other legumes, they are rich in lysine (18.4–20.4 mg/kg) but low in sulfur-containing amino acids, ranging from 3.8–4.3 mg/kg in cysteine and 2.5–2.8 mg/kg in methionine [43]. Interestingly, grass pea was found to be the only known dietary source of l-homoarginine, which has been shown to provide benefits in cardiovascular disease treatments. Therefore, as a nutraceutical, grass pea is an excellent example of a potential “functional food” [45,46]. This legume was found to be an excellent source of phenolics [47]. Despite its high nutritional value, grass pea consumption is still limited, likely because of its high content of the neuroexcitatory β-N-oxalyl-l-α, β- diaminopropionic acid (β-ODAP), which causes the neurodegenerative disease called neurolathyrism if overconsumed [48]. However, food processing, such as boiling, was shown to reduce β-ODAP concentration (down to 30% after 90 min boiling) [49]. Over the last few decades, several breeding programs tailored to produce grass pea varieties at reduced β-ODAP content were put in place, and different cultivar with a β-ODAP content <0.1%, have been released [44]. However, the stability of such varieties needs to be assessed over the long term because of the prime influence of climatic and edaphic conditions, along with the strong genotype × environment effect on the final β-ODAP content [50,51,52]. From an agricultural point of view, grass pea shows resistance to harsh environmental conditions [53], such as drought, heat, soil infertility, floods, and many ranges of biotic stresses, and minimal inputs and cost are required for its cultivation. Because of its valuable agronomic and nutritional properties, grass pea figures as a sustainable and nutritional legume. Therefore, its reintroduction into the human diet as a potential functional food would be desirable. As for industrial application, grass pea protein-based films and coatings for the food industry are reported [54], whilst the use of grass pea proteins in food formulation is still limited, probably due to their anti-nutritional factors [55].

Lupinus is a large genus comprising around 200 species, among which four are cultivated in different world regions, namely *L. angustifolius* (narrow-leafed), *L. albus* (white), *L. luteus* (yellow), and *L. mutabilis* (Andean). Australia is the leading producer of lupin, followed by Poland, Russia, Morocco, and Germany. From 1999 to 2018, the total area of lupin cultivation decreased by about one-third from 1.48 to 0.98 Mha, and total production declined by nearly half from 2.1 to 1.2 Mt [56]. *Lupinus albus* was the main *Lupinus* species cultivated in the Mediterranean region, and it is considered a valuable nutritive source containing lower amounts of fat (~6%), a high number of essential amino acids, important dietary minerals, and higher protein (~40%) and dietary fiber (~28%) contents [57]. Due to comparable quantities of proteins with similar amino acid profile, lupin is considered a cheap alternative to other legumes, such as soybean. Interestingly, lupin was found additionally rich in phytochemical, such as importantly bioactive peptides, alkaloids, polyphenols, phytosterols, tocopherols, that make this legume flour a potential food ingredient, especially for bakery products [58]. As an implication of human health, the high fiber and the antioxidant, antihyperlipidemic, and anti-inflammatory activities of the phytochemical of lupin act against various chronic diseases [58]. In contrast to grass pea, the usage of lupin proteins to develop novel foods (e.g., cheese analogue; gluten-free pasta, cookies, and cakes) [59,60,61,62] or to improve food nutritional value was widely explored [61,63]. Lupin protein supplementation was also found to affect positively organoleptic and textural properties of foods [64].

Winged bean (*P. tetragonolobus*) is a vining plant commonly found in humid countries such as Sri Lanka and Malaysia [65]. Similar to other legumes, the winged bean can fix atmospheric nitrogen, thus promoting the soil fertility and production of other crops such as rice [66]. Although its cultivation to a large scale is limited because of the high variability and low yield [67], the winged bean could be considered a multipurpose legume in terms of agricultural and nutritional properties. From an agricultural perspective, its cultivation requires low external inputs, making it suitable to be cultivated in areas where water scarcity and hot temperatures are increasing because of climate change [17]. In addition, it could be considered a good source of nutrients. Indeed, apart from having a high protein content equivalent to the soybean, its seeds contain a good balance of amino acids, including lysine, which is poor in cereal-based diets [68]. Some minerals found in this plant have been reported to be higher than soybean, including thiamin, riboflavin, and niacin [69]. Winged bean seeds are also rich in oil, particularly unsaturated oil, which is rich in Vitamin E. In addition, all parts of the plant (except the stems) are edible, palatable, and nutritious [70]. The high nutritional value of this legume, combined with its ability to grow in harsh environmental/climatic conditions, make the winged bean a key functional food, also alternative to meat consumption, which cultivation could be implemented, especially in semi-arid and hot regions where the under nourishing diet affect people’s health. Despite its high nutritional value, studies reporting the supplementation of winged bean in food formulation are scarce [71,72,73]. Recently, Saloko et el. reported that the addition of the mixture 9%-winged bean flour: 6% konjac flour in corn noodles significantly affects the final content of water, protein, dietary fiber, fat, and calcium, as well as improving some technological and organoleptic properties of the food [71].

**Figure 2:**
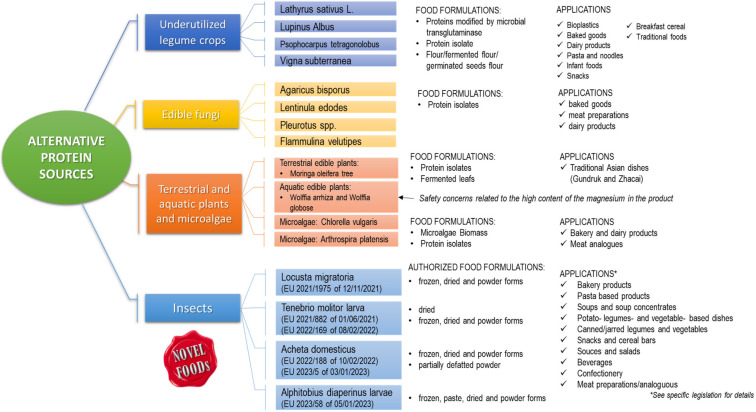
Food formulations and relative applications of different sources of proteins alternative to meat including underutilized legume crops, edible fungi, terrestrial, aquatic plants and microalgae, and insects.

The Bambara groundnut (*V. subterrenea*) is another underutilized legume with comparable potential content to soybean. This legume is indigenous to Africa, where it is considered the third most important leguminous crop after cowpea (*V. unguiculata*) and peanut (*Arachis hypogaea*) [74]. From a nutritional point of view, Bambara groundnut seeds contain 63% carbohydrate, 22% protein, and 6.6% fat [75], showing a total energy value greater than that of other common pulses, such as cowpea, lentil (*Lens esculenta*), and pigeonpea (*Cajanus cajan*). Bambara groundnut was found to be a good source of fiber, calcium, iron, and potassium, and among the different varieties consumed, the red seeds contain almost twice as much iron as the cream seeds, thus representing a good choice in areas where iron deficiency is a problem [75]. Because of its valuable nutritional value, Bambara groundnut was proposed as an ingredient to implement foods for infant weaning to improve dietary intakes of children in countries characterized by protein-to-energy malnutrition [76,77]. In addition to infant formulations, flour blends of Bambara groundnut were used for preparing high protein snack products. Snacks produced from blends of Bambara groundnut, cassava, and soybean at different levels reported protein content varying between 14.25% and 16.25%, of which the high protein content and acceptability was attributed to that containing 80% Bambara groundnut [78]. Using Bambara groundnut in enriching breakfast cereal and pasta, traditional foods and milk-based products are also reported [79]. Food applications of these alternative protein sources are given in Figure 2. The levels of starch and amylose (up to 53% of seed of starch and 15.7–35.3% of amylose in starch) and dietary fiber content (up to 10.3% of seed) make this crop suitable for the control of diabetes and high cholesterol correlated disease [80]. Similar to other legumes, Bambara groundnut shows high valuable agronomic properties, such as drought tolerant [81] and ability to grow in poor soils where most common crops do not thrive [82]. In the light of its nutritional and agronomic properties, the cultivation and consumption of Bambara groundnut has the potential to positively contribute to food and nutritional security at both the regional and global levels.

## 3. Edible Fungi

**Figure 3:**
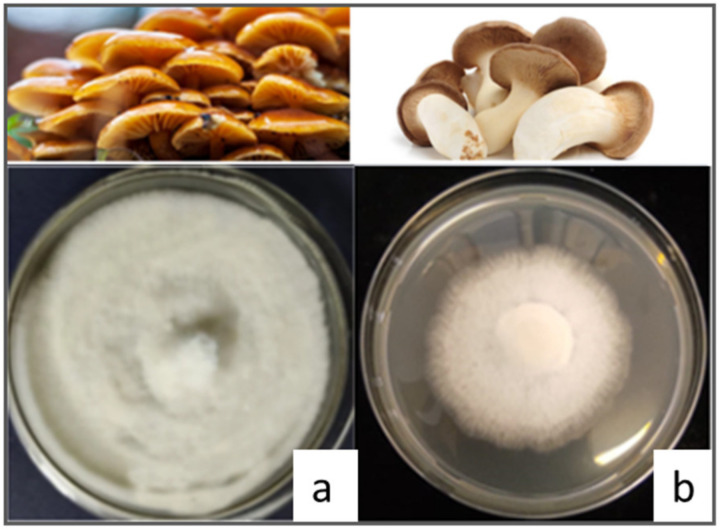
Edible fungi: panel (**a**) *Flammulina velutipes* grown on Potato Destrose Agar (PDA) for 12 days at 25 °C; panel (**b**) *Pleurotus eryngii* grown on PDA for 12 days at 25 °C.

Proteins from edible fungi are gaining enormous interest in their healthy nutritional profile. The most cultivated fungal species are *Agaricus bisporus* (button mushroom, white or brown, or portobello, representing about 40% of worldwide production), *Lentinula edodes* (about 25% of worldwide production), *Pleurotus* spp. (mostly *P. ostreatus*), and *Flammulina velutipes* [83]. Examples of *Pleurotus eryngii* and *Flammulina velutipes* grown on ad the appropriate substrate are reported in Figure 3.

Edible fungi have been widely studied as alternative sources of proteins; therefore, this section will provide an overall discussion. The amount of protein in edible fungi varies from 19 to 45% of dry matter (DM), depending on species, stage of maturation, parts of fungus, substrate, and technological process used for their cultivation [13]. The application of biotechnology (e.g., bio-refinery and solid state fermentation) in a bio-based circular economy to produce fungal proteins is encouraged and is being currently investigated [84,85]. The production of multiple secreted proteins or specific target proteins can be carried out using native strains or engineered by highly specialized fungal hosts that can perform various post-translational processing, making them more suitable than bacterial or yeast hosts [86]. Protein yields are also affected by the growth substrate; fungal cultivation on agro-industrial wastes (e.g., corn stover, paddy straw, peat extracts; ram horn hydrolysate) significantly increases the protein content, reaching a percentage higher than 45% DM [87]. Today, a derivative of *Fusarium venenatu*, an authorized protein source for sale by the UK Government since the 1990s, is produced to a large scale in a continuous fermentation process and exported globally under the brand name Quorn^TM^ [88]. Proteins from fungal dried powder (also named “mycoprotein”) are typically used to fortify various foods due to their high nutritional value. Fungal proteins are highly considered substituting partially or entirely animal-based protein foods such as meats [89]. Toxicology studies have ascertained that mycoproteins include no negative effects on the normal growth of humans and animals [90]. Amino acid analysis of *Pleurotus* spp. has demonstrated the presence of all essential amino acids [91], with leucine, aspartic acid, phenylalanine, and lysine being the most abundant. Umami amino acids were also found together with non-essential amino acids such as gamma- aminobutyric acid (GABA) and ornithine [92]. A recent study carried out on human volunteers has demonstrated that the biological value of proteins in mycoproteins is similar to milk proteins. The protein-to-energy ratio (PER) for nine species of edible mushrooms with a protein content from 16 to 37% was studied by Bach et al. in 2017 [93] and ranged from 0.051 to 0.098. These values were similar to those registered for foods such as beef jerky, whole milk, and lentils [13]. By considering the range of protein amount reported above, 100 g of dried mushrooms can cover from 29.41% to 66.00% of the Recommended Dietary Allowance (RDA) for men and from 35.80% to 80.35% for women [13]. Gonzalez et al. in 2020 [13] reported the True Protein Digestibility (TPD) for some edible mushrooms determined by using Sprague-Dawley rats as animal models. Values highlight a large variability (ranging from 42 to 80%) because of the different chemical composition, also within the same species, and probably because of conditions of cultivation or stage of the mushroom maturation. Less variable data are reported for the Biological Value (BV). In particular, 63.72, 71.94, 74.82 and 77.18 were reported for *Lentinus lepidus*, *P. sajor-caju*, *P. ostreatus,* and *L. edodes* proteins, respectively. These values showed that a high amount of amino acids is absorbed by the gut that is retained by the body [13], confirming the high nutritional value of mycoproteins. In addition to their nutritional properties, proteins from edible mushrooms have shown interesting biological activities that may improve health, treat and/or preventing disease [93]. Indeed, many proteins with interesting biological activities have been discovered and isolated from edible fungi, such as lectins, fungal immunomodulatory proteins (FIP), ribosome-inactivating proteins (RIP), antimicrobial or antifungal proteins, ribonucleases, and laccases [94]. Beneficial effects have also been attributed to mushroom-derived bioactive peptides [95]. Thus, several studies report the beneficial effects associated with mycoprotein ingestion [96,97,98]. New epidemiological work [98] studying mycoprotein-based food intake using data from the UK National Diet and Nutrition Survey shows that higher mycoprotein intakes are associated with significantly lower markers of glycaemia, improved fiber intake profiles, and diet quality scores. Thus, considering the beneficial effects, the use of mycoproteins has been reported in a wide variety of food products, including baked goods (such as pasta, breads, and cookies, refs. [99,100,101,102,103]), meat products [89], and dairy products [104], as shown in Figure 2. Beyond the nutritional value, improvements in physicochemical and/or organoleptic characteristics have also been reported. Recently, a recombinant Ery4 laccase, purified from *P. eryngii* and expressed in *S. cerevisiae,* was added to the manufacturing protocol of a dairy product, resulting in an increase in antioxidant properties and texture and protein content, with a product yield 10% higher than control sample [105,106]. This protein also proved to have the potential to degrade mycotoxin, contributing to increasing the safety standards of food products [107,108].

## 4. Terrestrial and Aquatic Plants and Microalgae

### 4.1. Terrestrial and Aquatic Plants

**Figure 4:**
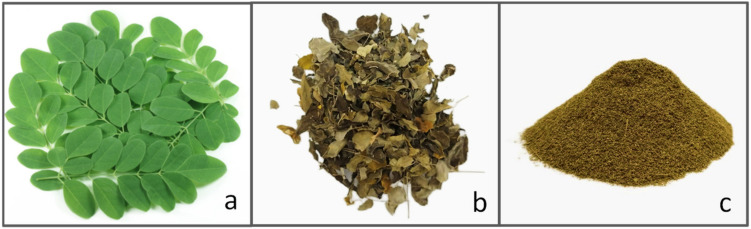
*Moringa oleifera* (**a**) fresh leaf (**b**) dried leaf (**c**) leaf powder.

Leaves can also represent a valuable source of plant proteins to be implemented in the formulation of meat alternative products. More than 70% of the proteins are in the chloroplast, where ribulose bisphosphate carboxylase (RuBisCO), an enzyme involved in photosynthesis, is the dominating protein [109]. The protein content of a leaf evolves during its development, resulting in a progressive degradation to free amino acids starting from the early senescence [109]. *Moringa oleifera* tree (also known as drumstick tree), and *Wolffia arrhiza* and *Wolffia globosa* (also known as duckweed), are only two examples of fully edible plants with a high protein content. However, the bio-accessibility of nutrients, particularly proteins, in vegetable foods is impaired by non-starch polysaccharides (cellulose, hemicellulose), polyphenols, and anti-nutritional factors (inhibitors of proteases and α-amylases) that interfere with food proteins and digestive enzymes [110]. The isolation of proteins from the vegetable matrices helps to improve the digestibility by removing fibers [111]. However, food-grade extraction procedures, including, for example, the two-step alkaline extraction/isoelectric precipitation, used for preparing protein isolates from seeds [112,113], fails when applied to leaf powder because of the low solubility of membrane proteins compared to seed storage proteins. *Moringa oleifera Lam* (*Moringaceae*) is a valuable perennial plant cultivated in tropical and subtropical regions. In Figure 4, an example of fresh *Moringa oleifera plant*, its dried leaf, and leaf powder to be employed in different preparations is depicted. *Moringa* is considered a superfood in India with several edible parts, including leaves, roots, seeds, bark, fruit, flowers, and immature pods. Being rich in proteins, vitamins and minerals, the leaves are part of the diet of pregnant women and weaning children with in vivo proved beneficial effects [114,115]. D’Auria et al. reviewed the nutritional profile and the food applications of the *Moringa oleifera* seeds and leaves [116]. The enzyme-assisted extraction of proteins from moringa leaf powder, using a cellulolytic enzyme mixture, by breaking down the matrix structure, made it possible to prepare a high protein concentrate (55.7%, *w*/*w*) that would not be achievable with alkaline extraction alone [117]. From a nutritional standpoint, the concentration process improved dramatically the in vitro digestibility of the *Moringa* leaf protein isolate by over 35% compared to the whole leaf powder, with a value (digestibility score of 99.9% and PDCASS 91.42%) that appears to be comparable to whey proteins and perfectly in line with the FAO requirements [117,118]. An alternative to protein isolation for improving the digestibility of leaves is the fermentation. Shi et al. [119] demonstrated that solid-state fermentation of drumstick leaf flour induced by *Aspergillus niger*, *Candida utilis* and *Bacillus subtilis* allowed to obtaining of high-quality proteins, increased concentrations of small peptides and amino acids, as well as reduced concentrations of anti-nutritional factors such as tannins, phytic acid, glucosinolates, and sugars. Overall, great interest is emerging towards the exploitation of *Moringa* as a value-added ingredient for both bakery products (e.g., bread, biscuit, cake, fresh and dried pasta, and snacks) and meat products [120] (See Figure 2). However, the supplementation of *Moringa* in the formulation of meat products still represents a partially explored research field, mainly when considering the bioaccessibility and bioavailability of *M. oleifera* bioactives, thus requiring future research works. It is important to highlight that plant extracts (such as those from *Moringa*) are recently gaining wide popularity in the meat industry since they are perceived by consumers as safe and included in the Generally Recognized as Safe (GRAS) category [121].

**Figure 5:**
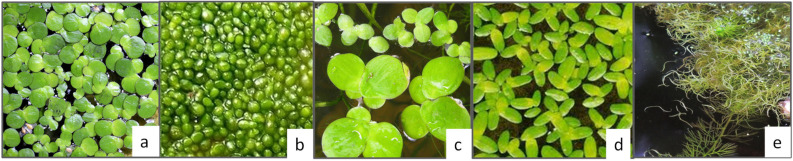
Duckweed genera: (**a**) Lemna minor, (**b**) Wolffia, (**c**) Spirodela polyrhiza, (**d**) Landoltia punctata, (**e**) Wolfiella gladiate.

While *M. oleifera* is a terrestrial panacea plant, duckweed is an aquatic superfood consumed in Asia [122]. It is a monocotyledonous plant of the family *Lemnacae*, including five genera (*Spirodela*, *Landoltia*, *Lemna*, *Wolffiella*, and *Wolffia*) shown in Figure 5, and belonging to the same genera as the Wolffia, which are aquatic [123]. On a dry base, the protein content of duckweed can be as high as 43%, including all essential amino acids [122]. The plant has been recently assessed for its safety as a food ingredient by EFSA in both forms, as powder [124,125] and as fresh vegetable, such as spinach [126]. The NDA panel of EFSA concluded that the high content of magnesium in the product would be of safety concern, and the safety of use of the novel products could not be established. However, the high protein content of the powder and its amino acid profile may push the use of protein isolates as an ingredient in the near future. The preparation of an alkaline extract from a *Lemna gibba* powder allowed the preparation of a 67.2% protein concentrate (*w*/*w*) with good solubility at pH higher than 6 and a similar gel strength at pH 4 to 7, comparable to soy proteins but lower than egg white proteins [127]. It is worth mentioning that the authors used a nitrogen-to-protein conversion factor of 5.8, as suggested by Martin et al. for the spinach RuBisCO, which is the most abundant protein in the leaf [128]. Protein isolates from *W. globose*, obtained using ultrasound-assisted extraction, also showed antimicrobial properties and better emulsifying stability compared to whey proteins for at least 24 h. Duckweed nutrient content and metabolite composition have gained extensive attention, particularly in the animal feed industry [129]. The application of duckweed as plant-based ingredient for future food products is very limited [129]. Figure 2 provides an overview of food applications described in the literature of alternative proteins extracted from these sources.

### 4.2. Microalgae

Microalgae are underwater aquatic plants that have been suggested as food ingredients for their high protein content ranging between 40–70%, depending on the species, poly-unsaturated fatty acids, and micronutrients (minerals and vitamins) [130,131,132,133]. Similar to other plants, microalgae synthetize RuBisCO, even though not to high levels as terrestrial and superficial aquatic plants such as the duckweed [134]. The essential amino acid profile of microalgae appears to be in line with those of soybean and egg proteins [133]. The mainstream microalgae explored as novel ingredients are *Chlorella vulgaris* and *Arthrospira platensis* also known as Spirulina. While *Chlorella* sp. is not classified as a novel food within the European Union, meaning they do not fall under Regulation (EU) 2015/2283 [34], Spirulina sp. is to be classified as a novel ingredient [135] and therefore requires a dedicated safety and nutritional assessment. Efforts have been made to look for technological approaches for improving the yield of productions through breeding and strain selection and genetic engineering [136]. Among the limitations associated with the use of whole microalgae biomass as the ingredient is the “grassy-fishy” flavor/aroma and the color [137,138]. Research efforts made possible the preparation of honey/yellow and the while Chlorella powders that are obtained from chlorophyll-free strains through the selection of low chlorophyll strains and the chemically induced random mutagenesis [139,140]. Both mutant strains of Chlorella have high protein content (40–50%) and essential fatty acids and are already a reality in the market commercialized by *Allmicroalgae*. The presence of the cellular walls, representing the 10% of the dry weight, is among the major obstacle to protein digestibility of the microalgae biomass. However, data on digestibility appears to differ depending on the species, from as high as 78% from the *A. platensis*, having a wall mainly composed of the peptidoglycan layer, to 45% *Nannochloropsis oceanica* and *Chlorella* [141,142,143]. Microalgae biomass has been used in the formulation of several food products from bakery to dairy products [138,144,145,146,147]. Despite the direct use of biomass would be economically preferable, cell walls and oil also have a negative impact on the texturing properties of the meat analogues prepared using microalgae [146,148]. The preparation of microalgae protein isolates follows a three-step approach with the mechanical or enzyme-assisted cell disruption, solubilization, and the concentration. Microalgae protein extracts are used for complementing formulations extruded for preparing meat analogues. Microbial proteins show excellent performances as functional ingredients, with excellent emulsifying and foaming properties with reduced dependency on pH and superior interfacial stabilization than animal proteins [149].

## 5. Insects

Insects are deemed a good source of micronutrients and proteins, with an average protein content of 40%, ranging from 20% up to over 70%, depending on the species [150]. Insects are a common food for 2 billion people in 119 countries across the globe. Over 2000 species are edible, and the most exploited as protein sources are *Coleoptera Beetles*, followed by *Lepidoptera Caterpillars*, *Hemynoptera*, wasps, bees, and ants. Although more familiar for Asiatic population, with a widespread consumption in India, China, Thailand, South Korea, Japan, but also Mexico, Brazil, and several African regions, insects are still novel foods for Western countries [20,21]. Due to the growing need for alternative sources of proteins and the attractive advantages shown in cultivating insects in terms of higher sustainability, such tendency of consuming insects-based foods is progressively revolutionizing Western eating habits and the European market. Three insect species that are widely bred in Europe (*Tenebrio molitor*, *Gryllodes sigillatus*, *Schisocerca gregaria*) are considered to have the biggest potential as food components since they contain the highest amount of protein estimated to be 52, 70, and 76%, respectively [151]. On the other hand, it has to be emphasized that often, the protein content can be overestimated due to the presence of a non-protein nitrogen [152]. Although insect proteins are characterized by high threonine and lysine content and low levels of methionine or tryptophan, the essential amino acids’ score for insects ranges from 46% to 96%, exceeding the lowest recommended level for human diets (>40%). The quantity of the same amino acids is even higher in insects than in plants and animals [153]. In addition, insect proteins are more digestible than plant proteins and exhibit lower digestibility than animal proteins [154]. Insect-based proteins are characterized by a low level of solubility ranging from 3% to 45%, which may be improved by enzymatic hydrolysis [155]. Therefore, the application of insect proteins is recommended for foods that do not require high solubility, such as meat analogs. Insect proteins generally have good oil and water retention capacity, emulsion activity, and foaming activity. Among them, edible grasshopper (*S. gregaria*) and honeybee (*Apis mellifera*) display highly emulsifying properties comparable to whey protein, showing their potential as alternative emulsifiers [156]. However, the functional characteristics of insects are different and strictly related to the species. For example, protein extracts obtained by conventional means such as *Hermetia illucens* and other insect species have poor solubility in aqueous media, and this may impair their nutritional value, biological activity, functionalities [157]. The proteins of *Protaetia brevitarsis* and *Allo-Myrina dichotoma* species exhibit higher protein thermal stability and emulsification activity than *T. molitor*, and, as result, they show better manufacturing characteristics [158]. On the contrary, the higher concentration of polyphenols in the protein concentrate of *Hermetia illucens* can reduce its emulsification activity [159]. Therefore, to increase the use of insect proteins in food production, the functional characteristics of insect proteins should be improved. Innovative processes have been optimized to increase specific abilities of insect proteins such as ultrasound [160], ultra-high pressure, pH-shifting technology [161], and cold atmospheric pressure plasma processing [162]. As already stated, the insects are considered novel foods within European countries and comply with specific regulations for the production, transportation, storage, and commercialization, both as animal feed and food. A blooming of European directives and regulations have been issued in the last 10 years in order to regulate the production and commercialization of novel foods as summarized in Table 1. In the last five years, the European Commission (EC) regulated the production, transport, and storage conditions of insect-based meal allowed in aquafeed from black soldier fly (BSF) (*Hermetia illucens*), common housefly (*Musca domestica*), yellow mealworm (*T. molitor*), lesser mealworm (*Alphitobius diaperinus*), house cricket (*Acheta domesticus*), banded cricket (*Gryllodes sigillatus*), field cricket (*Gryllus assimilis*), and Silkworm (*Bombyx mori*) [37,163]. In August 2021, the EC adopted the decision to allow the use of insect-processed animal proteins in formulated pig and poultry feeds [163]. The International Platform of Insects for Food and Feed (IPIFF)—namely the European umbrella organization representing stakeholders active in the production of insects for food and feed—is encouraging European member states to implement regulations from the European Commission [164], supported by favorable opinion of the EFSA Panel on Nutrition, Novel Foods, and Food Allergens (NDA) [165]. The commercialization of *Locusta migratoria* (EU 2021/1975 of 12/11/2021 [166]), *T. molitor* larva (EU 2021/882 of 01/06/2021 [167], EU 2022/169 of 08/02/2022 [168]), *A. domesticus* [EU 2022/188 of 10/02/2022 [169], EU 2023/5 of 03/01/2023 [22]), and *A. diaperinus* larvae (EU 2023/58 of 05/01/2023 [170]) as novel foods (under the frame of Regulation (EU) 2015/2283 [34]) has been authorized in specific formulations and applied to specific food categories listed in Table 2.

About house cricket, EFSA has highlighted that the consumption of the evaluated insect proteins may potentially lead to allergic symptoms. It was also noted that allergens present in the substrate fed to insects, when the whole insect is meant as the ingredient, may reach the ultimate consumers through the consumed insect [171]. The current market trends in modern sedentary lifestyles have driven the development of new functional products that can fulfill consumers’ demand for a healthy diet. Bread, in particular, is a vital staple food and is consumed throughout the world because it is rich in proteins but lacks some essential amino acids (lysine and trilysine) [172,173,174]. Innovative additives, such as insect flour, can be utilized in several bread formulations due to their high protein content, contributing to the enhanced quality and increased nutritional value of the final product [173,175]. For example, sourdough obtained from cricket powder hydrolysates has been reported to be rich in health-promoting molecules such as arachidonic acid and linolenic acid, which could be used for innovative bread production with high nutritional and functional value [176]. Previous studies have shown that wheat bread fortification with various edible insect flours, such as mealworm, buffalo worm, cricket [177,178], and the larvae of the black soldier fly [173,179] has improved the biological value of bread due to the high protein content characteristics. Pauter et al. [180] used 10% cricket powder to increase the protein content of muffins by 1.4-fold. In addition, da Rosa Machado and Thys [181] reported that the enrichment of cricket powder in gluten-free bread can lead to a final high protein content (8.53–12.52, wt%). The same trend was reported by Nissen et al. 2020 [182], where cricket powder was reported to provide gluten-free sourdough bread with high nutritional value proteins. In addition to the nutritional value, cricket enrichment helps products have a unique aroma [182] and improved texture, namely increased hardness and improved consistency [183]. Currently, more than 160 patents and around 500 articles have appeared in the literature about the applications of edible insects [184]. Figure 2 reports some main food applications referred to products fortified with insects in different authorized formulations.

## 6. Bioactive Properties of Non-Meat Proteins

Biologically active peptides (BPAs) are derived from proteins and exert a positive effect on humans due its health-promoting properties. They have a molecular weight lower than 6 kDa and contain between 3 and 20 amino acids residues that remain inactive in the parent protein and exhibit their activity after release and transport to the active site [185]. Structure–activity relationships are of importance, and the amino acid sequence of the peptide influences its biological activity. The presence of amino acids such as alanine, cysteine, histidine, lysine, leucine, methionine, proline, valine, tryptophan, and tyrosine may contribute to the antioxidant activity of peptides [186], whilst Pro, Lys, or aromatic amino acid residues contribute to Angiotensin Converting Enzyme (ACE) inhibitory activity [187]. BAPs can be released from food proteins during gastrointestinal digestion and through fermentation, germination, enzymatic hydrolysis, and during food processing. Novel processing technologies, such as high pressure, microwave, and pulsed electric field, have recently emerged to overcome the problems associated with the conventional hydrolysis methods [188]. Apart from the already mentioned above, BAPs exhibit a broad range of activities and can be involved in the gastrointestinal system (anti-obesity and satiety peptides), the cardiovascular system, the immune system (antimicrobial, antibiofilm, antiviral, cytomodulatory peptides), and the nervous system (opioid peptides) [189,190,191,192]. Most studied BPAs are derived from protein of animal origin [192]. However, alternative sources have been explored to discover novel sequences addressed to drug targets. Despite the bioactivity of several novel food ingredients described in the literature, the bioactive sequence is not known due to a lack of a tailored pipeline for the isolation and mass spectrometry mapping. On the other hand, protein hydrolysates are often mapped by mass spectrometry, and prediction of bioactivity is made based on bioinformatics, without an in vitro validation. Table 3 reports a list of active peptides from alternative sources. To the best of our knowledge, the reported sequences are the only identified ones since most studies involved protein hydrolysates and their partially purified fractions.

Enzymatically-derived BPAs from proteins of major legume species (soybean, cowpea, groundnut, common beans, pea, and chicken pea) display a wide spectrum of biological activities ranging from nutraceutical to therapeutic potential. The hydrolysis process occurs in vitro by using food grade enzymes (alcalase) or during food processing (fruit ripening process), the fermentation process (to obtain soy sauce, tempe, natto, and other fermented products), or the germination process (producing soybean sprouts, green bean sprouts, and sprouts products others) [248]. Except from lupin, peptides released from minor legume proteins still represent an unexplored field worthy to be deeply investigated. Lupin-derived peptides have shown opioid, immune-modulating effects, as well as the ability to reduce glucose, cholesterol, and blood pressure (antihypertensive), depending on the type of investigation (in vivo and in vitro) as depicted in Table 3. This last evidence suggests that these bioactive peptides could probably be useful nutraceutical components in the development of anti-diabetic foods [249]. Papain was found to be the most efficient protease for the production of winged bean seed hydrolysate with potent ACE inhibitory and antioxidant activities [250]. ACE inhibitory properties were also demonstrated in vivo by lowering blood pressure in rat models, fostering the potential of winged bean-derived peptides as a functional food ingredient [251]. Similar biological effects were showed by Bambara groundnut protein hydrolysate and their peptide fractions [252]. It was also demonstrated that BAPs produced from Bambara bean protein hydrolysates demonstrated antihyperglycaemic activity (dipeptidyl peptidase, DPP-IV, inhibitory activity), cardio-protecting effects by ACE inhibition, and resistance to simulated gastro-intestinal digestion [253]. Putatively, due to the high content of anti-nutritional factors and to the best of our knowledge, grass pea-derived peptides have not yet been reported. Proteins and peptides endowed with biological activity from edible fungi are widely reported (Table 3); several biological effects have been reported [254], which make this alternative source of meat promising for exploitation in the development of functional foods. To date, fungal cell factories for efficient and sustainable production of proteins and peptides endowed with technological and healthy properties represent a current challenge [86]. Likewise, edible fungi, *M. oleifera* seed or leaves protein hydrolysates and related purified peptides showed several beneficial effects, including antioxidant and anti-inflammatory [255], hypoglycemic [231], antimicrobial [232], antihypertensive [229] properties (see Table 3). Partially purified peptides fractions suitable for the regulation of hyperuricemia were also obtained from tryptic hydrolysate of *M. oleifera* leaves. These fractions contained the most abundant peptides TSIVGNV, ASGGIHV, GNAPGAV, AGEENAG, and VTPQPGVPPEEA, which after gastrointestinal digestion released di-/tripeptides responsible for the inhibition of xanthine oxidase, used as a target in the treatment of hyperuricemia [256]. Most studies also focused on the genus *Lemna*, although scant information is available on the biological and functional properties of the released peptides. Antimicrobial activity, ACE inhibitory, and DPPH scavenging activities were found for enzymatic hydrolysates obtained by *W. globose*, *Lemna minor*, and *Chlorella sorokiniana* proteins [257,258]. Microalgae peptides that mimic the functions of mediators involved in pathologic processes responsible for vascular damage playing a putative role in the prevention of cardiovascular disease were discussed by Li et al. [212]. Cancer-inhibiting or immunomodulating effects of microalgae have been reported, although the number of reports on bioactive peptide sequences is very limited [259]. These studies, antioxidant and ACE potential have been confirmed in vitro for other microalgae hydrolysates obtained from *C. vulgaris*, *Porphyra dioica*, *Fucus spiralis* [234,260,261]. Bioactive peptides with anti-cancer and immunomodulating activities have also been described in the literature for *C. vulgaris*, *C. pyrenoidosa* and *Dunaliella salina* [262,263,264,265]. The most commonly followed method of bioactive peptides generation from microalgae involves enzymatic digestion of microalgae protein concentrates followed by fractionation using milder techniques such as UF at different membrane pore sizes (3–30 kDa) followed by chromatographic purification. The consumption of insect proteins, silkworm pupa, has been associated with a reduced risk of many diseases, such as cardiovascular diseases (hypertension and hyperlipidemia), and cancer (hepatoma and gastric cancer) [266]. The release of bioactive peptides is suggested based on biological effects (Table 3). Indeed, silkworm pupa hydrolysate was reported to possess several bioactive properties, such as inhibiting ACE activity, antioxidant activity, immunomodulatory activity, improving hypercholesterolemia (Table 3), beyond a decrease in the allergenicity of silkworm pupa protein [267]. The antitumor activity of silkworm pupae protein hydrolysates was also investigated [268,269], although more evidence will be needed to validate these results. Techniques, such as ultrasound, have been applied to assist enzymatic hydrolysis and increase production of bioactive compounds [270]. One of the first and most frequently studied insect peptide hydrolysates was derived from the proteins of the species *Bombyx mori*. The ACE inhibitory properties were the predominant bioactivity determined. The ACE-inhibitory peptide sequences identified from protein of *B. mori* pupae were, for example, KHV, ASL, and GNPWM (Table 3). Peptides showing this activity were also identified in cricket (*Gryllodes sigillatus*), mealworm (*T. molitor*), and locust protein (*Schistocerca gregaria*) [271].

## 7. Safety Issues Linked to Emerging Proteins and Derived Products

Despite technological and industrial application, safety aspects of alternative proteins remain poorly described and tackled. Thus, in this section, we attempt to summarize their current food safety status, including the environmental impact and regulatory framework.

Despite their proven high nutritional value, minor/orphan legume crops consumption could represent a relevant risk for human health because of the intrinsic toxicity of some of these plants. As previously mentioned, the major nutritional problem of grass pea is a neurotoxic non-protein amino acid, b-N-oxalyl-L-a, b-diaminopropionic acid (β-ODAP) in its seeds. Over-consumption of these seeds as a staple food in an unbalanced diet for 3–4 months can cause a disease known as neurolathyrism, which manifests as paralysis of the leg muscles, muscular rigidity, and weakness in humans and domestic animals. The concentration of the β-form is about 95% of the total ODAP and its content in grass pea seeds strongly depends on environmental factors [272] and, by the variety cultivated, indeed a wide range of variation for ODAP content (0.02–2.59%) was observed in grass pea germplasm. Several efforts have been made in the past to reduce this neurotoxin to a safe level for human consumption spanning from the development of grass pea varieties with low ODAP to a low-cost agronomic practices and post-harvest processing [44]. Breeding programs aimed at combining low ODAP content with high valuable agronomic properties led to different grass pea lines with < 0.1% toxin content. Anyway, because of the prime influence of environmental conditions on ODAP concentration, more research tailored to investigate the performance of these genetic lines in field needs to be undertaken [273]. As for detoxifying processing, the combination of soaking and boiling were found to be the most promising strategy to reduce ODAP content up to 67% [44]. Very little information is available on the allergens of grass pea. In 2018, Xu et al., identified an abundant 47 kDa protein in grass pea, which further studies demonstrated to have a homology to the 47 kDa vicilin from pea and Len c 1 from lentil. Interestingly, this grass pea 47 KDa protein was recognized by immunoglobulin E (IgE) antibodies from sera of several patients allergic to peanut, leading to supposed cross-reactivity between grass pea and peanut [274]. Lupine is another orphan legume able to cause severe intoxications (e.g., trembling, shaking, excitation, and convulsion) on humans if over-consumed. This critical safety issue is mainly ascribed to the high content of quinolizidine alkaloids especially in lupine bitter species. These last show a general content of alkaloids ranging from 1 to 3% in the dry weight of their seeds, among which the lupanine, a molecule belonging to quinolizidine alkaloids, that represents the major component. Other minor quinolizidine alkaloids found were albine, hydroxylupanine, sparteine, anagyrine, lupinine, and angustifolin. The high content of quinolizidine alkaloids group was found associated with severe intoxication [61]. Despite its potential toxicity, bitter lupines are traditionally consumed after soaking and cooking that can reduce the content of alkaloids [275]. To protect consumer’s health, the health authorities of several countries fixed the maximum limit of alkaloids in lupine below 200 mg/kg [276]. In line with this recommendation, modern breeding programs focused on developing lupine varieties with low-alkaloid content, the so-called “cultivated sweet” lupine varieties [277]. Sweet lupine seeds show a mean total alkaloid content ranging from 0.3 to 0.5% in the dry weight of their seeds, depending on the species, geography, and climate. Sweet lupines preserve the good agronomic properties of wild lupin, such as high adaptability to temperate and cold climates, low-fertile soils, harsh conditions, and high nitrogen fixation ability [277,278,279], demonstrating high suitability for low-input agriculture. Lupine allergenicity represents another critical issue to be taken into consideration while suggesting this legume consumption. The recent FAO/WHO expert consultation of food allergy, because of the lack of data on potency and prevalence, excluded lupin from the list of priority allergens, making it a problem for a few regions [280]. It has been reported in fact that lupine sensitivity is more prevalent in specific areas, including Mediterranean countries and Australia, where it is more consumed, and where severe allergy symptoms have been recorded, especially for cross-reactivity with peanut and other legumes (lentils, peas, and soybean) [281]. According to the European legislation, the European Union Regulation No. 1169/2011 included lupine among the 14 ingredients that must be declared on food labels because it can trigger allergies or intolerances [282]. According to the Allergen Nomenclature Subcommittee of the World Health Organization and International Union of Immunological Societies [283], the following proteins globulins (α-, β-, and γ-conglutin), 2S albumins (δ-conglutin), and some minor fractions, such as pathogenesis-related (PR)-10 proteins (Lup a 4 and Lup l 4), nonspecific lipid transfer proteins (nsLTP) (Lup an 3), and profilins (Lup a 5) represent the main lupine allergens currently identified [281]. Different strategies have been put in place for reducing lupine allergy, including enzymatic hydrolysis and thermal treatments, such as boiling, autoclaving, microwave heating and extrusion cooking with promising results obtained for enzymatic hydrolysis [284] and prolonged autoclaving [285]. Similar to other legume seeds, winged bean and Bambara groundnut contain several anti-nutritive factors (ANFs), which could limit the consumption of these foods. Tannins, lectins, flatulence factors, phytoglutenins, saponins, and cyanogenic glycosides reduce the nutritional value of winged bean [286], while phytates and tannins are the main ANFs of Bambara groundnut. ANFs are renowned for affecting the bioavailability of nutrients and minerals because of chelation reactions that could occur during digestion, reducing the level of nutrients potentially absorbable [287]. Tannins and phenolic compounds, not specifically, could inhibit enzyme activity and interact with food proteins to form complexes by reducing their quality [288]. However, different processing methods have been put in place to eliminate safely these substances without reducing their nutritional composition. For example, the use of moist heat (such as autoclaving and boiling) or soaking has been shown to significantly reduce the ANFs in winged bean, while preserving high value nutrients [289]. Fermentation of the seeds and other processing methods could be successfully used for reducing ANFs content in Bambara groundnut as reported in some studies [290].

The main food safety hazard associated with mycoproteins is allergens. The ingestion of Quorn^TM^ -based foods caused allergic reactions (hives and anaphylaxis) in individuals with a history of mold allergies; the IgE-mediated allergy to mycoprotein may be caused by the acidic ribosomal protein P2 [291]. Despite their nutritional values, microalgae may also carry harmful substances, such as heavy metals and toxins, particularly because of contaminations. Although lower than allowed levels, certain food supplements derived from *Spirulina* spp. and *Chlorella* spp. contained cadmium, mercury and this was likely due to the lack of quality control measures [292].

Toxins in microalgal dietary supplements were found when the environment is contaminated with toxin-producing cyanobacteria; in particular, the production of toxins (such as anatoxin-a, cylindrospermopsin, microcystins and saxitoxins), may result from direct toxin production by microalgae (*Aphanizomenon flos-aquae*) or cross-contamination with other toxin-producing cyanobacteria. Contamination by pathogenic microorganisms and IgE cross-reactivity poses additional safety issues associated with microalgal food products [291].

Using insects as food ingredients involves different levels of risk: biological, chemical, and allergenic risk [293]. Legal documents ensuring safety of insects-based foods and feed are chronologically reported in Table 1. About the biological risk, processing of the insects (e.g., hot slaughtering and cooking) can help moderate and eliminate the microbiological risk [294]. Contamination by chemicals is of enormous concern as reported by EFSA [35], with specific regard to heavy metals (cadmium, mercury, lead, and arsenic) and the accumulated pollutants from the environment (e.g., hormones and pesticides) [295]. A crucial key step in controlling chemical risks relates to the breeding stage and transformation process [296]. In addition to biological and chemical risks, the allergen risk of triggering a severe reaction in sensitive consumers is basically due to the presence of tropomyosin and arginine kinase—two major proteins common in different insects and responsible for allergic reactions [32,297,298]. These are renowned pan-allergens known for their cross-reactions with homologous proteins of crustaceans and dust mites [299]. The results reported by De Marchi et al. [26] revealed that tropomyosin identified in cricket was the most significant IgE binding protein, and cross-reactivity of it with the shrimp tropomyosin was verified by ELISA. Silkworm bodies contain allergens in their different growth stages (larva, pupa, moth) and their metabolites (silk, molting, feces) and 45 potential allergens were found in silkworms and some of them detected also in larvae, pupae, moths, silk, slough, and feces [300]. Thus, the risk of developing allergic reactions by eating insects poses an actual concern in allergic consumers. The results of the homology comparison showed that the allergens of silkworms were likely to cross-react with the allergens of *Dermatophagoides farinae*, *Aedes aegypti*, *Tyrophagus putrescentiae*, *Triticum aestivum*, etc. [300]. Based on closely related phylogenesis, the frequently reported IgE-binding cross-reactions between these allergens from different sources may cause unexpected cross-allergic reactions in susceptible individuals [51]. Due to the close taxonomic relationship between arthropods and crustaceans, patients allergic to house dust mites, shrimp, and mealworms should be cautious in eating insects [296,301]. The proper labeling of food containing insects is a correct measure to be implemented by food manufacturers to protect allergic consumers’ health [302].

Overall, alongside proper labeling, also the current digital tools and e-commerce platforms available facilitating access to food and selection from the virtual shelves should be implemented with information on the safety and functional properties of foods, in particular novel foods, in order to protect the consumers’ health including vulnerable ones [303].

## 8. Conclusions and Future Perspectives

The efforts of recent years in scouting for novel proteins sources alternative to meat and the increasing use of these non-meat proteins in the development of innovative foods proved to attract consumers for being healthy and eco-sustainable. The most exploited non-meat protein sources including algae, insects, fungi, and underutilized crops capable of growing also in harsh conditions were deemed promising because they are environmentally friendly and able to tackle the impact of the climate change worldwide. *Fabaceae* (*Leguminosae*) family is a source of seeds known as pulses, showing the best compromise between sustainability and nutritional value also including grass pea, lupine, winged bean, Bambara groundnut and are considered underutilized legumes with promising potentials, because of their hardiness, remarkable nutritional profiles, and desirable protein content, especially in their seeds with a good resistance to draught conditions. Edible mushrooms are rich in water and provide proteins, dietary fiber, chitin, vitamins (folate and B12), and some minerals. They are poor in fat and sodium and can be proposed as part of a well-balanced diet. Aquatic plants and microalgae are underwater aquatic plants, which have been suggested as food ingredients for their high, poly-unsaturated fatty acids and micronutrients (minerals and vitamins). Insects are deemed a good source of micronutrients and proteins with an average protein content of 40%. Over 2000 species are edible and the most exploited as protein sources are *Coleoptera Beetles*, followed by *Lepidoptera Caterpillars*, *Hemynoptera*, wasps, bees, and ants. Due to the growing need for alternative sources of proteins and the attractive advantages shown in cultivating insects in terms of the higher sustainability, such tendency of consuming insects-based foods is progressively revolutionizing western eating habits, having recently entered also the European market. To summarize, an increase in demand for plant-based protein is expected to be a consolidated trend in the coming years as the international community looks for new sources of proteins to meet the needs of a growing population. Due to the healthy properties described for these foods and the high abundance of bioactive peptides and phytochemicals promoting antioxidant defense and reducing inflammation, more consumers in future will turn to vegetarianism and veganism, which will also contribute significantly to the demand for such products. On another perspective, despite the recommendations to increase plant-food consumption in human diet, there have been some concerns raised about the potential presence of allergens or chemical contamination in the case of insects posing some safety hazard to consumers. On one hand, the high demand for novel and existing sustainable protein sources (e.g., legumes, insects, algae, and cultured meat) to replace animal-based sources is dramatically increasing. This change in protein consumption calls for future re-evaluation of the current methods to assess their quality and bioavailability, due to the fact that the food diet is massively changing. This fosters a deeper investigation in the future, including re-evaluating the two conventional scores currently in use: PDCAAS (protein digestibility-corrected amino acid score) and DIAAS (proteins indispensable amino acid score) because of their limitations. This is necessary in order to address plant and novel proteins’ quality.

## Figures and Tables

**Table 2:** Summary of the insect-based products whose placing on the market has been authorized within the European Union as novel food in specific formulations and food categories.

Species	Form	EU Regulation	Specified Food Category
*Locusta migratoria* (migratory locust)	frozen, dried and powder forms	[166]	Processed potato products; legumes-based dishes and pasta-based products; meat analogues; soups and concentrated soups; canned/jarred legumes and vegetables; salads; beer-like beverages, alcoholic drink mixes; chocolate confectionery; nuts, oilseeds, and chickpeas; frozen fermented milk-based products; sausages
*Tenebrio molitor larva* (yellow mealworm)	dried	[167]	Multigrain bread and rolls; crackers and breadsticks; cereal bars; dried-pasta-based products; pasta-based dishes (excluding dried puffed pasta); pizza and pizza-like dishes; dried stuffed pasta based products; pre-mixes (dry) for baked products; sauces; potato, legumes-based dishes; whey powder; meat analogues; soups and salads; chips/crisps; beer-like beverages; mixed alcoholic drinks; alcoholic drink mixes; chocolate confectionary; nuts, oilseeds and chickpeas; frozen fermented milk-based products; meat preparations
	frozen, dried and powder forms	[168]	
*Acheta domesticus* (house cricket)	frozen, dried and powder forms	[169]	Protein products other than meat analogues; bread and rolls; bakery wares, cereal bars, and stuffed pasta products; biscuits; pasta-based products (dry); soups and soup concentrates or powders; processed potato products, legumes- and vegetable-based dishes, and pasta- or pizza-based product; corn flour-based snacks; beer-like beverages, alcoholic drink mixes; nuts, oilseeds and chickpeas; sauces; meat preparations; meat analogues; chocolate confectionary: frozen fermented milk-based products
	partially defatted powder	[22]	Multigrain bread and rolls; crackers and breadsticks; cereal bars; pre-mixes for baked products (dry); biscuits; pasta-based products (dry); stuffed pasta-based products (dry); sauces; processed potato products, legume- and vegetable-based dishes, pizza, pasta-based dishes; whey powder; meat analogues; soups and soup concentrates or powders; maize flour-based snacks; beer-like beverages; chocolate confectionary; nuts and oilseeds; snacks other than chips; meat preparations
*Alphitobius diaperinus larvae* (lesser mealworm)	frozen, paste, dried and powder forms	[170]	Cereal bars; bread and rolls; processed and breakfast cereals; porridge; pre-mixes (dry) for baked products; dried pasta-based products; stuffed pasta-based products; whey powder; soups; cereal-, pasta-based dishes; pizza-based dishes; noodles; snacks other than chips

**Table 3:** Bioactive peptides from proteins alternative to meat.

PEPTIDE NAME/SEQUENCE	SPECIES/CULTIVAR	BIOLOGICAL ACTIVITY	REF.
Fungal species			
RLPSEFDLSA FLRA	*Pleurotus cornucopiae*	ACE inhibitory	[193]
RLSGQTIEVTSEYLFRH			
AHEPVK	*Agaricus bisporus*	ACE inhibitory	[177]
PSSNK			
RIGLF			
GQGGP	*Pholiota adiposa*	ACE inhibitory	[194]
VIEKYP	*Grifola frondosa*	ACE inhibitory	[178]
GQP	*Macrocybe gigantea*	ACE inhibitory	[195]
LSMGSASLSP	*Hypsizygus marmoreus*	ACE inhibitory	[196]
QLVP	*Ganoderma Lucidum*	ACE inhibitory	[197]
QLDL			
QDVL			
AHEPVK	*Pleurotus cystidiosus*	ACE inhibitory	[198]
GPSMR			
WALKGYK	*Tricholoma matsutake*	ACE inhibitory	[199]
LLVTLKK			
IISKIK			
ILSKLK			
LIDKVVK			
MQIFVKTLTG KTITLEVEES DDIDNVKAKI QDKEG	*Calvatia caelata*	Antiproliferative and	[200]
		Antimitogenic Activities	
Ubiquitin-like peptide	*Cyclocybe aegerita*	Antiproliferative properties	[201]
Cordymin	*Cordyceps militaris Cordyceps sinensis*	Antifungal activity, Anti-inflammation and Antioxidant effect	[202,203]
Agrocybin	*Agrocybe cylindracea*	Antifungal activity	[204]
Eryngin	*Pleurotus eryngii*	Antifungal activity	[205]
POP	*Pleurotus ostreatus*	Ribonuclease and translation-inhibitory activities	[206]
Pleurostrin	*Pleurotus ostreatus*	Antifungal activity	[207]
SU2 peptide	*Russula paludosa*	HIV-1 reverse transcriptase inhibitory activity	[208]
WALKGYK	*Tricholoma matsutake*	Antioxidant activity	[199]
PEMP	*Pleurotus eryngii*	Antioxidant, antitumor and immunostimulatory activities	[209]
WGC	*Inonotus obliquus*	Anti-thrombotic tripeptide	[210]
IPLH	*Morchella esculenta*	Activation of enzymes for alcohol metabolism	[211]
IPIVLL			
Edible insects			
KHV	*Bombyx mori*	ACE inhibitory	
ASL			[28]
GNPWM			
ProASN-Pro-ASN-THR-ASN	*Silkworm pupae*	Immunoregulatory activity	[212]
HPP	*Silkworm pupae*	Cholesterol biosynthesis inhibition	[213]
SGQR			
FKVPNMY	*Silkworm pupae*	Antioxidant activity	[214]
AVNMVPFPR			
VNMVPFPR			
Defensin		Antimicrobial peptides	[215]
Defensin-like peptide 1 (DLP1)	*Hermetia illucens*		
Defensin-like peptide 2 (DLP2)			
Defensin-like peptide 3 (DLP3)			
Defensin-like peptide 4 (DLP4)			
Cecropin			
Cecropin Z1			
Cecropin 1 (Hicec1)			
Cecropin-like peptide 1 (CLP1)			
Cecropin-like peptide 2 (CLP2)			
Cecropin-like peptide 3 (CLP3)			
Attacin			
Hermetia illucens-attacin			
Sarcotoxin			
Sarcotoxin 1			
Sarcotoxin 2a			
Sarcotoxin 2b			
Sarcotoxin 3			
Stomoxyn			
StomoxynZH1 (a)			
YKPRP	Cricket	ACE inhibitory	[216]
PHGAP			
VGPPQ			
TGAQSLSIVAPLDVLRQRLMNELN-RRRMRELQGSRIQQNRQLLTSI	Cricket	Diuretic activity	[217]
TAN	Mealworm	ACE inhibitory	[218]
NIKY			
AKKHKE	Mealworm	Hepatoprotective activity	[219]
Minor legumes			
LILPKHSDAD		Regulation of insulin and glucose metabolism through the inhibition of Dipeptidyl peptidase IV	[220]
GQEQSHQDEGVIVR			
LTFPGSAED	*L. angustifolius*	Hypoglycemic and hypolipidemic,	[220]
		ACE inhibitor activities	[221]
TFPGSAED	*L. angustifolius*	ACE-inhibitory activities	[222]
LTFPG			
YDFYPSSTKDQQS	Lupine	inhibition of 3-hydroxy-3-methylglutaryl CoA reductase (HMGCoAR) and the modulation of cholesterol metabolism in HepG2 cells	[223]
FVPY	Lupine	Antioxidant activity	[224,225]
GPETAFLR	*L. angustifolius*	Antioxidant and anti-inflammatory activities	[225]
IQDKEGIPPDQQR; AKIQDKEGIPPDQQR;	*L. Albus*	Anti-inflammatory activity	[226]
LIFAGKQLEDGR;			
LDDALRAEK; RRAIGK; RDDAASCLVR			
PSELSGAAH	*L. mutabilis*	Antioxidant activity	[227]
R.AVNELTFPGSAEDIER.L;	*L. albus*	ACE-inhibitors	[220]
K.ELTFPGSAEDIER.L;			
A.IPPGIPYWT.Y;			
E.LTFPGSAED.I;			
YPNQKV	Winged bean	ACE inhibitory and anti-oxidative activities	[228]
FDIRA	Winged bean	ACE inhibitory and anti-oxidative activities	[228]
Terrestrial and aquatic plants and microalgae			
LGF	*Moringa oleifera* leaf	ACE-inhibitory activities	[229]
GLFF			
GY, PFE, YTR, FG, QY, IN, SF, SP, YFE, IY, LY	*Moringa oleifera* seeds	Antioxidant activity	[230]
KETTTIVR	*Moringa oleifera* seeds	α-glucosidase inhibitory activity	[231]
MCNDCGA (MOp3)	*Moringa oleifera* seeds	Antimicrobial activity	[232]
HVLDTPLL (Mop2)	*Moringa oleifera* seeds	Antimicrobial activity	[233]
DYYKR	*Porphyra dioica*	ORAC activity	[234]
YYIA	*Porphyra dioica*	ACE inhibitory activity	
YLVA	*Porphyra dioica*	DPP-IV inhibitory activity	
VEEY	*C. ellipsoidea*	ACE inhibitory peptide	[235]
VECTGPNRPQF	*Chlorella vulgaris*	antioxidant, antiproliferative, anti-inflammatory	[236,237,238]
LLAPPER	*Pavlova lutheri*	Anti-cancer activity	[239]
MGRY	*Pavlova lutheri*	Anti-cancer activity	[240]
AFL, FAL, AEL, VVPPA	*Chlorella vulgaris*	ACE-I inhibitory activity	[241]
IAE, FAL, AEL, IAPG, VAF	*Spirulina platensis*	ACE-I inhibitory activity	[241]
RLVNDSHRLATGDVAVRA	*Spirulina platensis*	Antibacterial peptide	[242]
PNN	*Spirulina platensis*	Antioxidant activity	[243]
YGFVMPRSGLWFR	*Spirulina platensis*	Anticancer peptide	[244]
IQP	*Spirulina platensis*	ACE-I inhibitory activity	[245]
TDP[I/L]AAC[I/L]	*Spirulina platensis*	Iron-chelating activity	[246]
FRESSAPEQHY	*Spirulina platensis*	ACE-I inhibitory activity	[247]

## Data Availability

Not applicable.
